# Distraction Osteogenesis of Severely Resorbed Mandible

**DOI:** 10.1155/2022/7651439

**Published:** 2022-01-13

**Authors:** Salah Sakka, Ali Al Rafedah, Nasser Alqhtani, Adel Alenazi

**Affiliations:** Department of Oral and Maxillofacial Surgery and Diagnostic Sciences, Prince Sattam Bin Abdulaziz University, Al-Kharj, KSA, Saudi Arabia

## Abstract

Edentulous patients require an adequate rehabilitation so that the alveolar ridge in the interforaminal region be restored for subsequent implant-supported overdentures. The ultimate goal of distraction is to reconstruct the alveolar ridge to a suitable height and width compatible with Atwood class 2 in an appropriate direction allowing the sagittal interalveolar relation to be normally restored. *Methods*. A 65-year-old man presented with a grade 4 Mandibular atrophy as per Atwood classification which resulted in unsatisfactory treatment with full dentures. Endo-Distractor Krenkel® device was used for anterior mandibular vertical distraction osteogenesis. Four mandibular implants (ITI Straumann, Basel, Switzerland) measuring diameter *Ø* = 4.1 mm and length *L* = 16 mm were inserted after the required retention period. Standardized prosthetic treatment was completed with titanium bar retained over dentures. *Results*. A distraction of 11 mm was achieved within 18 days followed by a retention period of 4 months. No signs of infection nor distractor anchorage loosening were detected, and minimal lingual tilting has occurred. *Conclusion*. Distraction is possible on severely atrophic mandibles. The quality of bone reconstruction is satisfactory for both functional and esthetic results.

## 1. Introduction

Severely resorbed mandibular alveolar ridges are always being a genuine challenge for dentists to attain an adequate oral rehabilitation. Dental implant surgery techniques have been successfully used for the treatment of such dilemma of severe atrophy [1]. However, serious complications were also associated with such treatment approach [2, 3].

In 1992, the concept of distraction osteogenesis was pioneered in oral and maxillofacial surgery for lengthening of the human mandible by gradual distraction [4]. Several clinical cases were then reported [5–7]. Many of the related devices are bulky and anchored by two miniplates which require removal in a second procedure.

In 2009, the Endo-Distractor Krenkel® was presented [8]. The screw of this simple device is anchored in the center of the bony arch and the osteotomized alveolar segment. It gently separates the bony segments gradually to a carefully chosen distance in the decided direction.

In this paper, we present a case of an elderly male patient with severe mandibular alveolar ridge. The Endo-Distractor Krenkel® was used for bone regeneration in order to support a subsequent implant placement.

## 2. Case Description

A 65-year-old man of nonsignificant medical history presented with a chief complaint of unsatisfactory conventional treatment with full dentures, resulting in severe atrophy of the alveolar ridge. Class 4 Mandibular atrophy was graded according to Atwood classification [9] using preoperative lateral transcranial X-rays. The patient was evaluated clinically and by orthopantomography and cephalograms after the distraction and before placing the implants.

**Figure 1:**
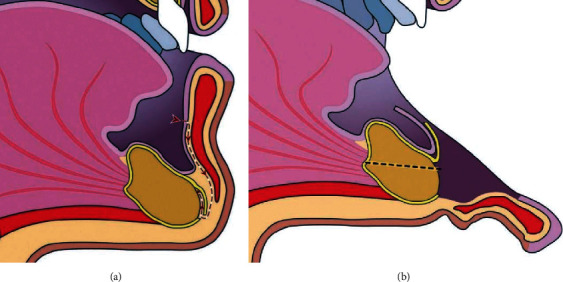
Schematic drawing showing (a) soft tissue preparation technique and (b) deflected tissues after dissection and the osteotomy line.

Surgery was done under general anesthesia because of the danger of bleeding in the floor of the mouth. A fully vascularized osteotomy surface is an essential precondition for an optimal distraction osteogenesis, and that was achieved by supraperiosteal dissection of the osteotomy segment. The dissection of the soft tissues was a modified Edlan-Mejchar technique [10]. The only difference was in creating a superior pedicle periosteal flap for coverage of the osteotomy gap (Figures 1(a) and 1(b)).

**Figure 2:**
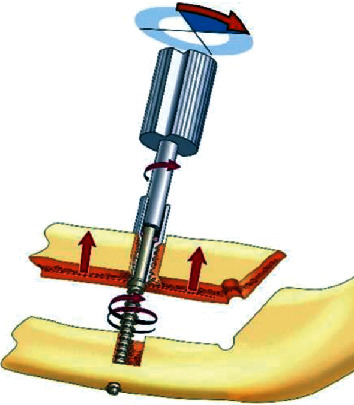
Schematic drawing and split image of the Endo-Distraction Implant Krenkel® and its biomechanical function.

The next would be the identification of the mental nerves for the lateral extension of the osteotomy. A bone height of 8 mm should be available to obtain segments of 4 mm each. Then, the osteotomy was performed parallel to the lower edge of the mandible (mandibular plane) using a W&H surgical microsaw-blade shape handpiece. According to the planned direction, a tap hole (*Ø* = 2.0 mm) was prepared in the body of the basal segment for the right-hand threaded distraction screw. In the osteotomy segment, a larger tap hole (*Ø* = 3.2 mm) was prepared for the left-hand threaded hollow screw. The distraction screw protrudes through the tap hole of the hollow screw 5 mm above the alveolar crest for the use of the screwdriver. The threaded distraction screw was covered by the threaded hollow screw, which is permanently anchored in the marginal bone segment (Figure 2).

The right-hand thread of the distraction screw and the left-hand thread of the hollow implant guarantee the stable fixation of the hollow implant when gradually unscrewing the threaded screw during distraction. The primary osteotomy gap of 3 mm would help in the initial thickness of callus formation.

**Figure 3:**
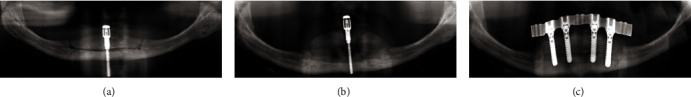
Orthopantomography follow-up. (a) X-ray performed after the placement of the Endo-Distraction device with an initial gap between the osteotomized segments. (b) X-ray showing bone formation after 6 months of retention period. (c) Posttreatment X-ray with fully functional implants connected by a bar.

After surgery, the Endo-Distraction Implant Krenkel® was safeguarded and blocked with a cover screw during the first retention time (Figure 3(a)). The resultant callus formation would predispose for the anticipated distraction osteogenesis.

The distraction period would start postoperatively after the first retention time for 7 days. The ends of the threaded screw are square headed, and each thread has a lead of 1 mm for one full turn.

The patient or one of his relatives did the distraction, exactly as instructed by the surgeon, on an “at home” basis with weekly controls.

The number and the amount of the daily extension movements was increased according to the thickness and stretchability of the newly formed distraction callus. The initial start was for 0.25 mm once a day for the first 8 days which allowed a “soft start.” This was necessary for a safe osteogenesis without creating fresh bleedings within the osteotomy gap bearing in mind that the daily distraction distance should not exceed more than one tenth of the momentary distraction gap. The twist movements would be increased up to 2 × 0.25 mm a day for the next 6 days followed by a period of 3 × 0.25 mm for 4 days. Thus, a distraction of 11 mm was achieved within 18 days. At the end of the distraction period, the Endo-Distraction Implant Krenkel® is again safeguarded and blocked by the cover screw for the second retention period of 4 months (Figure 3(b)).

No signs of infection nor distractor anchorage loosening were detected, and minimal lingual tilting has occurred. The amount of tilting was assessed by lateral transcranial radiographs with the distraction screw postoperatively and at the end of the retention period.

Perfect preoperative oral hygiene and oral mouth rinsing with chlorhexidine as well as treating with systemic antibiotics for three days are mandatory to prevent the infection.

**Figure 4:**
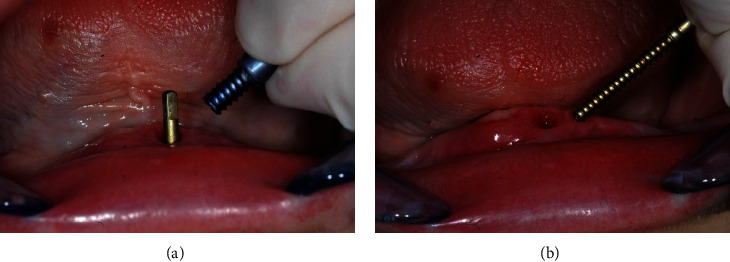
(a) Simple removal of the left-hand threaded hollow screw. (b) Easy removal of the right-handed distraction screw leaving a tiny hole in the mucosa, which subsequently healed.

The device was then retrieved and easily unscrewed as the screws were not firmly osseointegrated (Figures 4(a) and 4(b)).

The patient was scheduled for insertion of 4 mandibular implants (ITI Straumann, Basel, Switzerland) measuring diameter *Ø* = 4.1 mm and length *L* = 16 mm.

Standardized prosthetic treatment was performed with titanium bar retained over dentures. Additional 10-15 mm distal extensions lead the force distributed to the interforaminal implants, protecting the highly atrophic molar region (Figure 3(c)).

## 3. Discussion

The most important indication for this technique of distraction osteogenesis is a mandible with severe Atwood class 4 to 6 atrophy [11]. It primarily aims to rebuild the alveolar ridge to an adequate height and width consistent with Atwood class 2 in an appropriate direction to restore the sagittal interalveolar relation to normal.

The vertical bone gain obtained by distraction may reach 15 mm in a more “physiological” way than the vertical guided bone regeneration (GBR), without the need for bone grafting, and features less morbidity [12].

Vertical alveolar distraction osteogenesis is not an uncomplicated procedure, and complications can range from fractures of basal bone, fracture of transport segment, breakage of distractor, and lingual displacement of the distracted segment [13]. Conventional distraction devices require secondary surgery for the removal of plates, with repeated dissection of the mental muscles which may jeopardize the esthetic outcome. On the other hand, no need for secondary surgery for removal of the Endo-Distractor device used since the threaded distraction screw and the threaded hollow screw are visible in the oral cavity and the retrieval instruments can be easily used. Lingual tilting of the proximal bone segment is a related common complication of the conventional devices that requires secondary bone grafting before implant placement [14]. In contrast, lingual tilting of the distraction device used in this clinical report was negligible. Moreover, temporary dentures are no longer recommended during the retention time to prevent overloading the system that may induce anchorage loosening of the distraction device [8].

Special attention should be paid to blood supply, especially in the proximal segment, to prevent necrosis and infection. Terminal branches of the sublingual arteries should also be preserved to avoid hemorrhage during dissection and implant placement in the interforaminal region [15]. No other postoperative complications were observed like anchorage loosening of the distractor device, osteomyelitis, mandibular fracture, and implant failure.

## 4. Conclusion

The results show that alveolar distraction is possible on severely atrophic mandibles. The quality of bone generated was satisfactory for both functional and esthetic results. Surgical difficulty was lesser than with conventional distraction techniques, and the rate of complications was minimal.

## Data Availability

Patient's informed consent for publication of this case report and accompanying images to support the findings of this study are available from the corresponding author upon request.
